# Interaction between Attention and Bottom-Up Saliency Mediates the Representation of Foreground and Background in an Auditory Scene

**DOI:** 10.1371/journal.pbio.1000129

**Published:** 2009-06-16

**Authors:** Mounya Elhilali, Juanjuan Xiang, Shihab A. Shamma, Jonathan Z. Simon

**Affiliations:** 1 Department of Electrical and Computer Engineering, Johns Hopkins University, Baltimore, Maryland, United States of America; 2 Starkey Laboratories, Eden Prairie, Minnesota, United States of America; 3 Department of Electrical and Computer Engineering, University of Maryland, College Park, Maryland, United States of America; 4 Institute for Systems Research, University of Maryland, College Park, Maryland, United States of America; 5 Department of Biology, University of Maryland, College Park, Maryland, United States of America; Newcastle University Medical School, United Kingdom

## Abstract

The mechanism by which a complex auditory scene is parsed into coherent objects depends on poorly understood interactions between task-driven and stimulus-driven attentional processes. We illuminate these interactions in a simultaneous behavioral–neurophysiological study in which we manipulate participants' attention to different features of an auditory scene (with a regular target embedded in an irregular background). Our experimental results reveal that attention to the target, rather than to the background, correlates with a sustained (steady-state) increase in the measured neural target representation over the entire stimulus sequence, beyond auditory attention's well-known transient effects on onset responses. This enhancement, in both power and phase coherence, occurs exclusively at the frequency of the target rhythm, and is only revealed when contrasting two attentional states that direct participants' focus to different features of the acoustic stimulus. The enhancement originates in auditory cortex and covaries with both behavioral task and the bottom-up saliency of the target. Furthermore, the target's perceptual detectability improves over time, correlating strongly, within participants, with the target representation's neural buildup. These results have substantial implications for models of foreground/background organization, supporting a role of neuronal temporal synchrony in mediating auditory object formation.

## Abstract 2

Bottom-up (stimulus-driven) and top-down (attentional) processes interact when a complex acoustic scene is parsed. Both modulate the neural representation of the target in a manner strongly correlated with behavioral performance.

## Author Summary

Attention is the cognitive process underlying our ability to focus on specific aspects of our environment while ignoring others. By its very definition, attention plays a key role in differentiating foreground (the object of attention) from unattended clutter, or background. We investigate the neural basis of this phenomenon by engaging listeners to attend to different components of a complex acoustic scene. We present a spectrally and dynamically rich, but highly controlled, stimulus while participants perform two complementary tasks: to attend either to a repeating target note in the midst of random interferers (“maskers”), or to the background maskers themselves. Simultaneously, the participants' neural responses are recorded using the technique of magnetoencephalography (MEG). We hold all physical parameters of the stimulus fixed across the two tasks while manipulating one free parameter: the attentional state of listeners. The experimental findings reveal that auditory attention strongly modulates the sustained neural representation of the target signals in the direction of boosting foreground perception, much like known effects of visual attention. This enhancement originates in auditory cortex, and occurs exclusively at the frequency of the target rhythm. The results show a strong interaction between the neural representation of the attended target with the behavioral task demands, the bottom-up saliency of the target, and its perceptual detectability over time.

## Introduction

Attention is the cognitive process underlying our ability to focus on specific components of the environment while ignoring others. By its very definition, attention plays a key role in defining what foreground is, i.e., an object of attention, and differentiating it from task-irrelevant clutter, or background [1]–[5]. In the visual modality, studies have shown that figure/ground segmentation is mediated by a competition for neural resources between objects in the scene [2],[6],[7]. This competition is biased in favor of different objects via top-down attention as well as behavioral and contextual effects that work to complement or counteract automatic bottom-up processes. An intricate neural circuitry has been postulated to take place in this process spanning primary visual, extrastriate, temporal, and frontal cortical areas [7]–[19].

In the auditory modality, however, there have been a limited number of studies that attempted to explore the neural underpinnings of attention in the context of auditory stream segregation, and the mechanisms governing the extraction of target sounds from a background of distracters [20]–[28]. It is largely unknown how top-down (e.g., task-driven or context-dependent) and bottom-up (e.g., acoustic saliency or “pop-out”) attentional processes interact to parse a complex auditory scene [29],[30].

**Figure 1:**
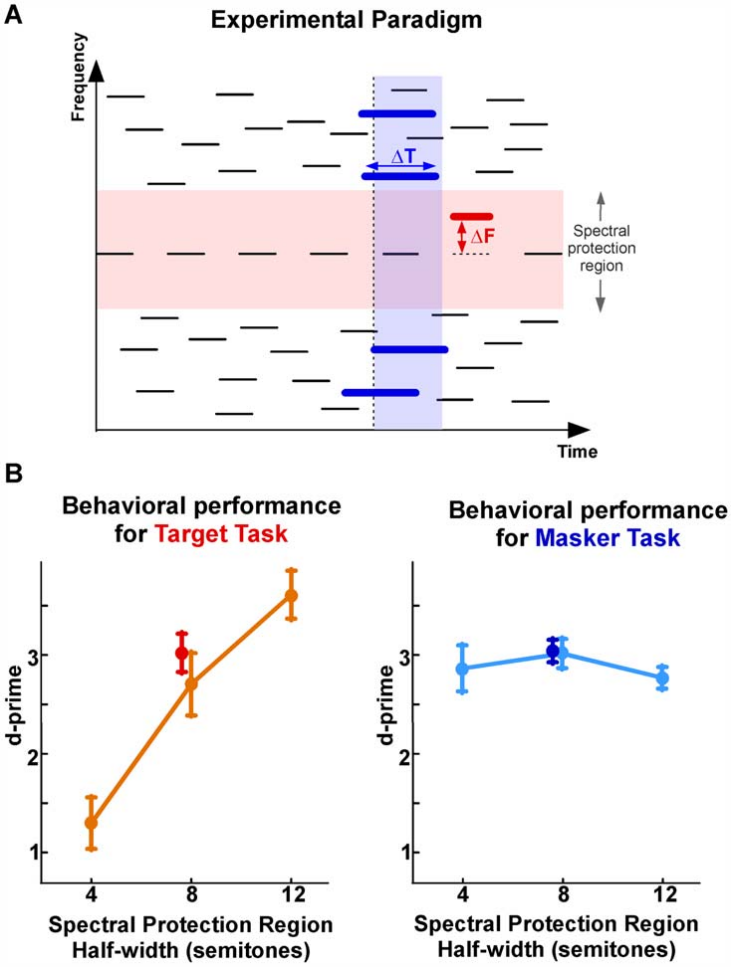
Stimulus description and behavioral performance. (A) Cartoon spectrogram of a typical stimulus. The stimulus consists of a repeating target note embedded in random interferers. A spectral protection region surrounds the target frequency with a spectral width of twice the minimal distance between the target note and nearest masker component (orange band). In the target task, participants were instructed to detect a frequency-shifted (ΔF) deviant in the repeating target notes. In the masker task, participants were instructed to detect a sudden temporal elongation (ΔT) of the masker notes. (B) Behavioral performance results for target and masker tasks, as measured by d-prime as a function of spectral protection region width. Orange (respectively, light-blue) lines show the mean performance in task detection in the target task (respectively, masker task) in the psychoacoustical study. Red (respectively, dark-blue) points show the mean performance in task detection in the target task (respectively, masker task) in the MEG study (eight-semitone condition only). Error bars represent standard error.

In a simultaneous behavioral and neurophysiological study using magnetoencephalography (MEG), we illuminate this interaction using stimuli shown in Figure 1A, consisting of a repeating target note in the midst of random interferers (“maskers”). This design generalizes paradigms commonly used in informational masking experiments, [31], which explore how listeners' ability to perceive an otherwise salient auditory element is strongly affected by the presence of competing elements. For these stimuli, the ability to segregate the target note depends on various acoustic parameters, including the width of the spectral protection region (the spectral separation between target and masker frequencies). We adapt classic informational masking stimuli to the purposes of this study by randomly desynchronizing all background maskers throughout the duration of the trial, making the target note the only regular frequency channel in the sequence (with repetition rhythm of 4 Hz). The informational masking paradigm has been shown to invoke similar mechanisms to those at play in classic stream segregation experiments [32],[33], both in the systematic dependence of performance on the size of masker–target spectral separation, as well as the improvement of performance over time over the course of few seconds.

While maintaining the same physical stimulus, we contrasted the performance of human listeners in two complementary tasks: (1) a “target task” in which participants are asked to detect a frequency-shifted (ΔF) deviant in the repeating target signal; and (2) a “masker task” in which participants are asked to detect a sudden temporal elongation (ΔT) of the masker notes. Crucially, attention is required to perform either task, but the participants' attention must be focused on different sound components of the acoustic stimulus in each case. Additionally, all the stimuli are diotic (identical in both ears), averting any confounding effects of spatial attention.

## Results

The effect of spectral protection region width on the performance of both tasks is illustrated in Figure 1B. In the left panel, it can be seen that the detectability of the target becomes easier with increasing protection region (significantly positive slope; bootstrap across participants, *p*<10^−4^), a result that is in line with previous hypotheses of streaming that correlate the ease of target detection with the frequency selectivity of neurons in the central auditory system [34]–[38].

In contrast, the same manipulations of protection region do not substantively affect masker task performance (right panel) (not significantly different from zero; bootstrap across participants, *p*>0.3). The masker task, designed to divert attentional resources away from the target, involves a more diffuse attention to the spectrally broad and distributed masker configuration; and compared to the target task, reflects a different top-down bias in the way the same stimulus is parsed. The behavioral performance was unchanged whether tested under purely psychoacoustic or neural recording conditions (no significant difference; unpaired *t*-test; target task: *t* = −0.75, *p* = 0.46; masker task: *t* = 0.09, *p* = 0.93).

For the neural recordings, we used the stimuli with the eight-semitones spectral protection region because they roughly matched the behavioral performance across tasks (d-prime for both is approximately equal to three). The target task is not at ceiling with the chosen protection region, hence still engaging participants' selective attentional processes.

**Figure 2:**
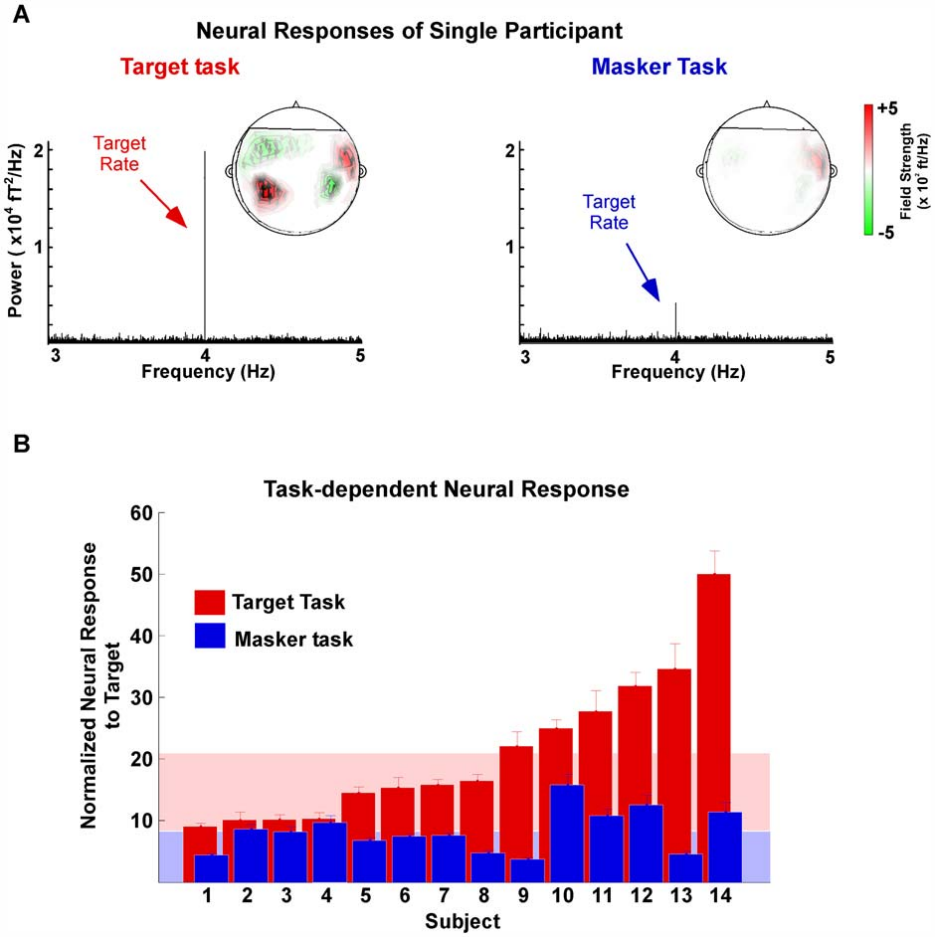
Neural responses. (A) Power spectral density of MEG responses for a single participant (participant 14 in Figure 2B below) in target (left) and masker (right) tasks, averaged over 20 channels. Insets: the MEG magnetic field distributions of the target rhythm response component. Red and green contours represent the target magnetic field strength projected onto a line with constant phase. (B) Normalized neural response to the target rhythm by participant (individual bars) and task (red for target task, blue for masker task). The normalized neural response is computed as the ratio of the neural response power at the target rate (4 Hz) to the average power of the background neural activity (from 3–5 Hz; see Materials and Methods). Bar height is the mean of the 20 best channels; error bars represent standard error. Light-pink background (respectively, light-blue) is the mean over participants for the target task (respectively, masker task).

Depending on listeners' attentional focus, the percept of an auditory target in a complex scene is differentially mirrored by the responses of neurons in auditory cortex. Using the high temporal resolution of MEG, we measure the neural responses to this stimulus paradigm in 14 human participants. Figure 2A reveals that, during the performance of the target task, the target rhythm emerges as a strong 4-Hz component in the neural signal of an individual participant. In contrast, during the masker task, the cortical response entrained at 4 Hz is noticeably suppressed in comparison (Figure 2A, right panel). This differential activation is strong evidence of the modulatory effect of task-dependent attention on the neural representation of a single acoustic stimulus, much like visual attention [39],[40]. Additionally, this attentional effect on the neural signal is not just momentary but is sustained over the duration of the trial (steady state).

This attentional effect is confirmed in the population of 14 participants (Figure 2B), with an average normalized neural response of 20.9 in the target task and 8.3 in the masker task: a gain of more than two and a half for neural phase-locked, sustained activity when participants' attention is directed towards the repeating note (individually, 11 out of 14 participants showed a significant increase: paired *t*-test, *p*<10^−4^). Direct correlation between the target task neural response and target task behavior is not observed, but as shown below, changes in a participant's target neural response are significantly correlated with changes in the participant's behavioral responses.

The MEG magnetic field distributions of the target rhythm response component, examples of which are shown in the inset of the graphs in Figure 2A, reveal the stereotypical pattern for neural activity originating separately in left and right auditory cortex. The neural sources of all the target rhythm response components with a sufficiently high signal-to-noise ratio originate in auditory cortex [41]. The neural source's mean displacement from the source of the auditory M100 response [42] was significantly different (two-tailed *t*-test; *t* = 2.9, *p* = 0.017) by 13.8±4.9 mm in the anterior direction, for the left auditory cortex only (no significant differences were found in the right hemisphere due to higher variability there). The displacement was not statistically significant in the remaining directions (3.2±3.5 mm lateral; 11.3±6.4 mm superior); the goodness of fit for these sources was 0.51±0.05 (artificially reduced in accordance with [43]). Assuming an M100 origin of planum temporale, an area of associative auditory cortex, this is consistent with an origin for the neural response to the target rhythm in Heschl's gyrus, the site of core auditory cortex including primary auditory cortex, and a region known to phase-lock well to 4-Hz rhythms [44].

**Figure 3:**
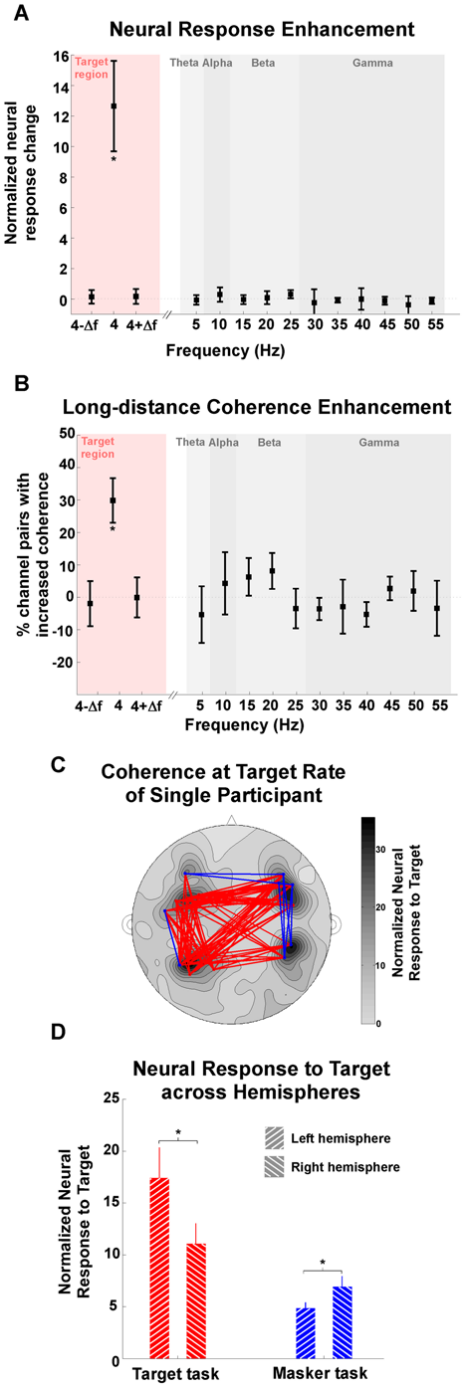
Power and phase enhancement during target task. (A) Normalized neural response of target task relative to masker task shows differential enhancement exclusively at 4 Hz (the frequency of the target rhythm). Each data point represents the difference between normalized neural response of target relative to masker task; error bars represent standard error The asterisk at 4 Hz shows that only that particular frequency yields a statistically significant enhancement. (B) Phase coherence between distant MEG channels of target relative to masker task. The difference between the number of long-range channel pairs with robust increased coherence in target task, and channel pairs with decreased coherence, is normalized over the total number of long-range channel pairs. The phase enhancement is significant (shown with asterisk) only at 4 Hz. (C) Channel pairs with robust coherence difference at target rate for single participant, overlaid on the contour map of normalized neural response at target rate. Each channel pair with enhancement coherence is connected by a red line, whereas pairs with decreased coherence are connected by a blue line. Coherence is only analyzed for the 20 channels with the best normalized response to target rhythm. (D) Neural responses to target across hemispheres. The 20 channels with the strongest normalized neural response at target rate were chosen from the left and right hemispheres, respectively, to represent the overall neural activity of each hemisphere. Neural responses were averaged across the 20 channels, and 14 participants were compared across hemispheres and tasks. The left hemisphere shows stronger differential activation at target rate in target task, whereas the right hemisphere shows stronger activation in masker task (asterisks indicate that the differences are significant).

The neural response change at the target rate of 4 Hz is highly significant (bootstrap across participants, *p*<10^−4^) (Figure 3A). In contrast, there is no significant change in normalized neural response at other frequencies, whether at frequencies nearby (one frequency bin on either side of 4 Hz) or distant (alpha, theta, and low gamma band frequencies sampled with approximately 5-Hz spacing up to 55 Hz). This demonstrates that this feature-selective sustained attention modulates the cortical representation of the specific feature, but not general intrinsic rhythms, whether in the same band or other bands.

Changes in response phase coherence across channels were also assessed at the same frequencies (Figure 3B, sample participant in Figure 3C). This analysis focuses on the distant channel pairs with enhanced phase coherence at each specific frequency. Only the phase coherence at the target rate shows a significant enhancement (bootstrap across participants, *p* = 0.002), further demonstrating that change from one form of attention to another does not modulate general intrinsic rhythms. This 30% enhancement is distributed across channel pairs, revealing increased phase coherence both within and across hemispheres.

We also observe a task-dependent hemispheric asymmetry in the representation of the neural response at the target rate. During the target task, the left hemisphere showed a greater normalized neural response than the right hemisphere (bootstrap across participants, *p* = 0.001); during the masker task, the right hemisphere showed a greater normalized neural response than the left hemisphere (bootstrap across participants, *p* = 0.04) (Figure 3D).

**Figure 4:**
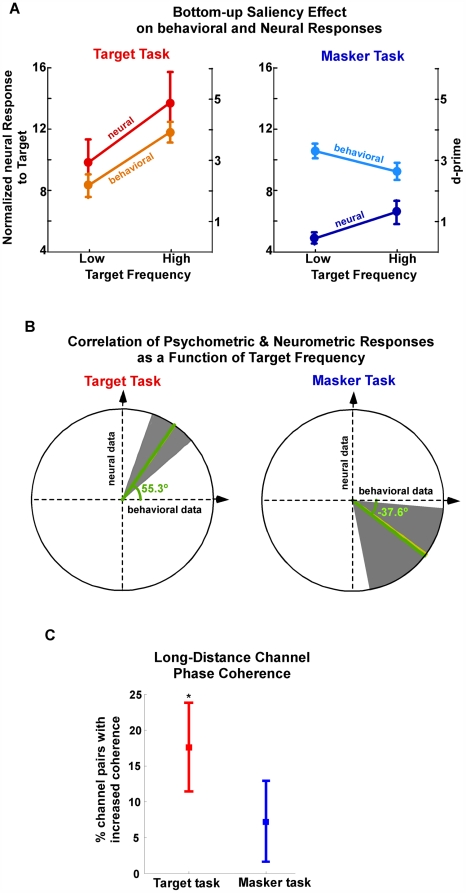
The effect of bottom-up acoustic saliency on behavior and neural responses. (A) Normalized neural response to target rhythm, and behavioral performance, as a function of target frequency in target task (left) and masker task (right), averaged over participants. Error bars represent standard error. (B) Correlation of behavioral and neural responses as a function of target frequency. The ratio of the neural to behavioral response differences as a function of target frequency, interpreted as a slope angle, is averaged across participants yielding a mean slope angle of 55.1° for target (left) task and −36.3° for masker (right) task (yellow line). Bootstrap estimates (overlying green lines) and their 95% confidence intervals (gray background) confirm the positive (respectively, negative) correlations for target (respectively, masker) task. (C) Phase coherence between distant MEG channels of target relative to masker task for high-frequency targets over low-frequency targets. High- versus low-frequency targets show significant enhancement only for target task (indicated by the asterisk).

Together with the behavioral demands of the task, the bottom-up saliency of a target note contributes to both the neural response and participant performance. A close examination of the physical parameters of the stimulus reveals that the frequency of the target note affects the audibility of the repeating rhythm, with higher-frequency targets popping out more prominently than their lower-frequency counterparts. This variation in the pop-out sensation may be explained by the contours of constant loudness of human hearing showing an approximately 5-dB increase over the target note range 250–500 Hz [45], because our stimuli were normalized according to their spectral power, not loudness. We exploit this physical sensitivity of the auditory system and determine the effect of this target pop-out on the neural and behavioral performances in both target and masker tasks. Figure 4A (orange line) confirms that behavioral performance in the target task is easier for higher-frequency targets (>350 Hz) than for lower frequencies (*t*-test; *t* = −3.3, *p* = 0.002). Correlated with this trend is an increased neural response to the target for higher frequencies compared to lower frequencies (red line) (increase not statistically significant alone). Conversely, the masker task shows a trend of being oppositely affected by the physical saliency of the target note despite its irrelevance for task performance (approaching significance; *t*-test, *t* = 1.8, *p* = 0.08). On the one hand, the neural power is increased for high-frequency targets reflecting their increased audibility (dark-blue line) (though not statistically significant alone). On the other hand, as the target becomes more prominent, the participants' performance of the background task deteriorates, indicating a distraction effect caused by the presence of the repeating note (light-blue line). Additionally, phase coherence is significantly enhanced for high-frequency targets over low-frequency targets only during the target task (bootstrap across participants, *p*<10^−3^) (Figure 4C). This result confirms that the physical parameters and acoustic saliency of a signal can interfere with the intended attentional spotlight of listeners and effectively deteriorate task performance [46],[47], both neurally and behaviorally.

In order to establish the correspondence within participants between the neural and behavioral responses under both task conditions in a parametric way, we quantified the slope (converted into an angle) relating the normalized neural signal with the listener's d-prime performance on a per-participant basis. The average slope angle for the target task is 55.1°, i.e., a positive slope, demonstrating the positive correlation between the two measures. Bootstrap analysis confirms this; Figure 4B, left panel, illustrates both the bootstrap mean of 55.3° (green line) and the 5th to 95th percentile confidence limits (gray background), all with positive slopes. Analysis of the masker task also demonstrates the anticorrelation trend between the neural and behavioral data, with an average slope angle of −36.3° shown in yellow. The bootstrap analysis also confirms this; Figure 4B (right panel) shows that the 5th to 95th confidence intervals (gray background) yield a robust negative slope with a bootstrap mean of −37.6° (green line).

**Figure 5:**
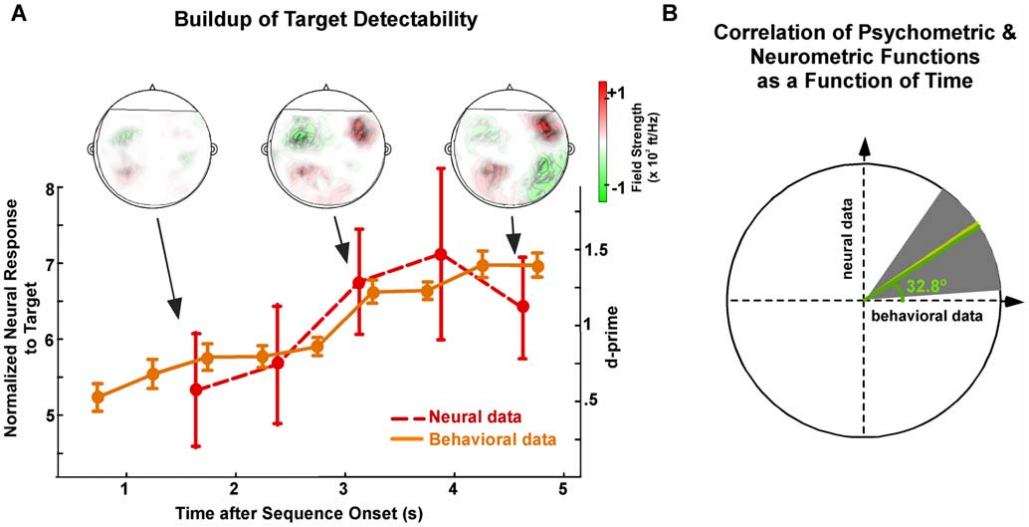
Buildup over time of behavioral and neural responses in target task. (A) Normalized neural response to target rhythm, and behavioral performance, as a function of time in target task, averaged over participants. Error bars represent standard error. Insets: the MEG magnetic field distributions of the target rhythm response component for a single participant at representative moments in time (participant 10 from Figure 2B). (B) Correlation of behavioral and neural responses as a function of time. The ratio of the neural to behavioral response trends as a function of time, interpreted as a slope angle, is averaged across participants, yielding a mean slope angle of 34.3° (yellow line). Bootstrap estimates (overlying green line) and the 95% confidence intervals (gray background) confirm the positive correlation between the psychometric and neurometric buildup curves.

The perceptual detectability of the regular target rhythm improves over time, following a pattern that is highly correlated with the neural buildup of the signal representation. Consistent with previous findings of buildup of auditory stream segregation [24],[35],[48],[49], participants' performance during the target task improves significantly over several seconds as shown in Figure 5A (solid orange line) (bootstrap across participants, *p*<10^–4^). This similarity suggests that target detection is mediated by top-down mechanisms analogous to those employed in auditory streaming and object formation [50]. These streaming buildup effects tend to operate over the course of a few seconds, and cannot be explained by attentional buildup dynamics reported to be much faster or much slower in time [51],[52]. Moreover, the neural response to the target rhythm also displays a statistically significant buildup (Figure 5A, dashed red line) (bootstrap across participants, *p* = 0.02) closely aligned with the behavioral curve, and consequently, decoupled from the actual acoustics. The remarkable correspondence between these two measures strongly suggests that the enhanced perception of the target over time is mediated by an enhancement of the neural signal representation, itself driven by an accumulation of sensory evidence mediated by top-down mechanisms. No such neural buildup of the neural response to the target rhythm is present for the masker task.

The MEG magnetic field distributions of the target rhythm response component in Figure 5A (insets), showing the stereotypical pattern of neural activity originating separately in left and right auditory cortex, illustrate the changing strength of the neural activity over time in an individual participant.

We confirm the correlation within participants between the psychometric and neurometric curves over time by running a bootstrap analysis on a per-participant basis. As expected, the slope correlating the d-prime and neural response curves for each participant yield a mean positive slope angle of 34.3°; bootstrap across participants shows a mean of 32.7°, with the 5th to 95th confidence intervals falling within the upper-right quadrant (Figure 5B).

**Figure 6:**
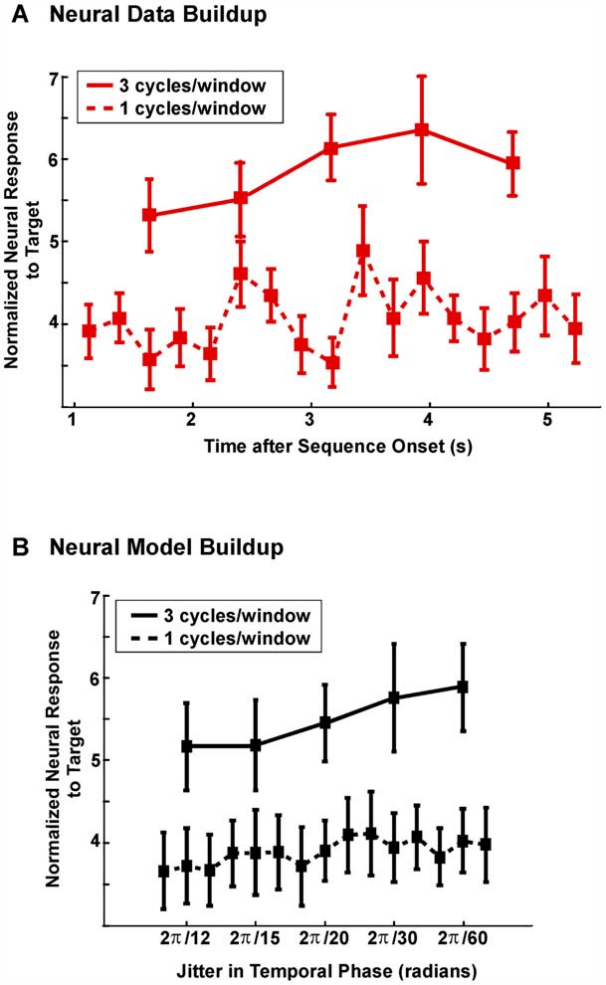
Analysis of neural buildup over time in target task for different duration windows, both for data and in a model of the data. (A) Normalized neural response to target rhythm as a function of time, in target task, averaged over participants. The solid curve is identical to the red curve in Figure 5. The dashed curve is the result of identical analysis except that the normalized neural response is calculated for every 250-ms cycle of the target rhythm, rather than over the 750-ms window of three-cycles used above. Only the longer window shows buildup, implying that it is not power per cycle that is growing, but phase coherence over several cycles. Error bars represent standard error over all participants. (B) Model results for 750 ms (three cycles) windows, solid curve, and for 250 ms (one cycle) windows, dashed curve. The modeled normalized neural response rises as temporal phase jitter decreases, but only in the three-cycle case, since the power per cycle is constant but the temporal phase coherence across cycles increases. Error bars represent standard deviation over 30 simulation runs.

We also note that the subsegments over which the neural buildup is measured are required to span several rhythmic periods (at least three; see Figure 6A). There is no buildup using intervals with shorter durations, despite sufficient statistical power. (This can be shown via the data plotted in the dashed curve in Figure 6A. The normalized responses in the range 3.5 to 4.5 are elements of an *F*(2,180) distribution, corresponding to *p*-values in the range 1.5% to 3.5%.) This implicates temporal phase coherence (in contrast to spatial phase coherence) as critical to the buildup of the neural target representation. That is, the power in each period is not increasing, but the power integrated over several periods is increasing. This can only occur if the phase variability decreases with time, i.e., the neural buildup is due to a buildup in temporal phase coherence rather than power.

As noted above, the subsegments, or windows, over which the neural buildup is measured are required to span at least three rhythmic periods, since there is no buildup observed using intervals with shorter durations. Figure 6A illustrates this buildup for both the three-cycle and one-cycle cases. The requirement of a longer time window shows that the buildup is not merely due to increased power at 4 Hz, since in that case, a window of one rhythmic period would also show buildup. This in turn implies that temporal phase coherence (in contrast to spatial phase coherence) is critical to the buildup of the neural target representation.

This is further demonstrated by a quantitative model. Typical simulated response profiles generated by the model are shown in Figure 6B. The horizontal axis in the model is not increasing time, but decreasing variability of the distribution of phase of the 4-Hz signal (i.e., the phase of the signal has greater variability initially and gets more regular as one proceeds along the axis). In the three-cycle window case, the buildup is pronounced, but not in the one-cycle window case. Note that the model does not attempt to emulate the downturn at the end of the experimental curve, nor does it attempt to emulate the rate at which the buildup occurs as a function of time (which would assume a linear decrease in temporal phase coherence over time).

The model results show that buildup can be due to increasing temporal coherence, and not due to increasing power. The neural noise, representing the stochastic firing patterns of the neurons underlying the MEG signal, is also required for the model's agreement with the data. A slight rise in the model's one-cycle window case may be seen, but it is not due to power (which never changes), rather it is due to increased coherence over trials, which is a weak side effect of increased temporal coherence.

## Discussion

This study's novel experimental paradigm builds on previous work in stream segregation using simpler stimuli [34],[35],[53],[54], but (1) using a richer stimulus design and (2) keeping the physical parameters of the stimulus fixed while manipulating only the attentional state of the listeners. One major finding is that auditory attention strongly modulates the sustained (steady-state) neural representation of the target. Specifically, sustained attention correlates with a sustained increase in the time-varying neural signal, in contrast with onset transients [22],[55] or nonspecific, constant (“DC”) [56]–[58] effects of attention on auditory signals. The location of the modulated neural representation is consistent with core auditory cortex, hence supporting current evidence implicating neuronal mechanisms of core auditory cortex in the analysis of auditory scenes [35],[59]–[61]. Furthermore, this modulation of neural signal is significantly distant from the source of the M100 and so cannot be explained as simply a train of repeated M100 responses. This steady-state increase in the signal strength is specific to the frequency of the target rhythm, and is additionally complemented by an enhancement in coherence over distant channels, reflecting an increased synchronization between distinct underlying neural populations. This attentional effect (in both power and phase) appears exclusively at the target frequency and is absent not only from other frequency bands whose intrinsic rhythms and induced response might show attentional changes, but even from adjacent frequency bins, which argues against any theory of neural recruitment or redistribution of energy at the low-frequency spectrum. Therefore, our findings argue that processes of attention interact with the physical parameters of the stimulus, and can act exclusively to enhance particular features to be attended to in the scene, with a resolution of a fraction of a hertz. Our analysis focuses on steady-state components of feature-based analysis, hence, complementing event-based analyses that relate temporal components of the recorded potential to specific mechanisms of feature-based attention [62]–[66].

Second, the data reveal that enhanced acoustic saliency (driven by bottom-up processes), which causes an increase in perceptual detectability, also correlates with an increase in the sustained power and coherence of the neural signal. In this case, the increase in neural signal occurs regardless of the task being performed, but with different behavioral consequences: in the target task, it leads to an increase in performance, but in the masker task, a decrease (via interference). This outcome allows us to give different explanations of this “attentionally modulated” neural change: as a marker of object detectability during the first task, but as a neural correlate of perceptual interference during the second task.

Third, the data show a left-hemisphere bias in the cortical representation of the target, for the target task, suggesting a functional role of the left hemisphere in selective attention, consistent with previous findings in visual [67] and auditory [61],[68] modalities. This bias may also be due to a left-hemisphere bias specific to Heschl's gyrus (the location of core auditory cortex), for slow rhythmic tone pips (without a masker background), as seen in [69],[70]. In contrast, for the masker task, the hemispheric bias in cortical representation of the (now nonattended) target is reversed to the right, and might be simply due to the nature of the attentional demands of the task (more diffuse attention to the global structure of the sound), or to the right-hemispheric bias of steady-state responses when attention is not specifically directed to the rhythm [71]. It also appears, for both tasks, that the deviant detection itself is not guiding the lateralization of the response, running counter to that of Zatorre and Belin [72], since the task/deviant requiring spectral change detection shows a left-hemisphere bias, and the task/deviant requiring temporal change detection shows a right-hemisphere bias.

Finally, this study offers the first demonstration of the top-down–mediated buildup over time of the neural representation of a target signal that also follows the same temporal profile of the buildup based on listeners' detectability performance in the same participant. Using the current experimental paradigm, we are able to monitor the evolution in time of attentional processes as they interact with the sensory input. Many studies overlook the temporal dynamics of the neural correlates of attention, either by using cues that prime participants to the object of attention (thereby stabilizing attention before the onset of the stimulus), or by explicitly averaging out the buildup of the neural signal in their data analysis (focusing instead on the overall contribution of attention in different situations, and not monitoring the dynamics by which the process builds up). Our findings reveal that even though the sensory target signal is unchanged, attention allows its neural representation to grow over time, closely following the time course of the perceptual representation of the signal, within participants.

Together, these findings support a view of a tightly coupled interaction between the lower-level neural representation and the higher-level cognitive representation of auditory objects, in a clear demonstration of auditory scene segregation: the cocktail party effect [73]. Our experimental paradigm allows both task-driven (top-down) and stimulus-driven (bottom-up) processes to guide perception. For listeners performing the target task, the target rhythm is the attended auditory object, a foreground stream to be separated from a noisy background. The masker task, requiring the listener to reverse the role of the foreground and background, allows the contrasting situation to be considered under otherwise identical acoustical conditions. This permits a controlled de-emphasis of the auditory role of the target rhythm, without the need for a “passive” listening condition under which the amount of the listener's attention is lessened, but actually unknown, and strongly variable across participants.

The data suggest that new models of attention may be required, based on temporally coherent or locally synchronous neural activity rather than neural amplification [74]. The buildup of neural responses over time is seen only when integrated over several periods of the target rhythm, but not for individual periods. This result is difficult to explain using standard models of attention that rely solely on gain-based changes, or even on gain/spectral-sensitivity hybrid models [75],[76]. Instead, a more plausible theory of neural mechanisms underlying the role of top-down attention in the buildup of perceptual streams would involve top-down projections acting in conjunction with the physical stimulus as regulators or clocks for the firing patterns of neuronal populations in auditory cortex. Another conceivable mechanism for this increase in temporal coherence may arise from general sharpening of temporal tuning, which would work for auditory streams far more complex than the regular stream presented here. The neural underpinnings of this bottom-up/top-down interaction are likely to mediate changes in the response profiles of cortical neurons, via mechanisms of synaptic and receptive field plasticity which have been shown to be gated by attention; whereby attention plays a crucial role in shifting cortical circuits from one state to another depending on behavioral demands [77]–[80]. We speculate that temporal patterns of neuronal firings are crucial in any scene segregation task to resolve the competition between attended and unattended objects, hence, delimiting the cognitive border between different streams.

Overall, a significant outcome of this study is that it not only demonstrates a strong coupling between the measured neural representation of a signal and its perceptual manifestation, but also places the source of this coupling at the level of sensory cortex. As such, the neural representation of the percept is encoded using the feature-driven mechanisms of sensory cortex, but shaped in a sustained manner via attention-driven projections from higher-level areas. Such a framework may underlie general mechanisms of “scene” organization in any sensory modality.

## Materials and Methods

### Participants

Nine participants (six males; mean age 29 y, range 24–38 y) participated in the psychoacoustic study. Eighteen participants (11 males; mean age 27 y, range 21–49 y) participated in the MEG study. Three participants took part in both studies. Among the 18 participants in the MEG study, four participants were excluded from further analysis due to an excess of nonneural electrical artifacts or an inability to perform the tasks, leaving 14 participants (eight males; mean age 27 y, range 21–49 y). All participants were right handed [81], had normal hearing, and had no history of neurological disorder. The experiments were approved by the University of Maryland Institutional Review Board, and written informed consent was obtained from each participant. Participants were paid for their participation.

### Stimulus Design

The stimuli were generated using MATLAB (MathWorks). Each trial was 5.5 s in duration with 8-kHz sampling. Every trial contained one target note, repeating at 4 Hz, whose frequency was randomly chosen in the range 250–500 Hz in two semitone intervals. The background consisted of random tones at a density of 50 tones/s, uniformly distributed over time and log-frequency (except for the spectral protection region). The frequencies of the random notes were randomly chosen from the five-octave range centered at 353 Hz, in two semitone intervals, with the constraint that no masker components were permitted within a four, or eight, or 12 semitone around the target frequency (the spectral protection region half-width). This random sampling of masker frequencies ensures a minimum spectral distance of two semitones between maskers, and keeps the probability of harmonically related maskers minimal. Masker and target tones were 75 ms in duration with 10-ms onset and offset cosine ramps. All masker tones were presented at the same intensity as the target tone.

Fifteen exemplar stimuli were generated for each of the four condition types: null condition (no deviants); target condition (one target deviant per stimulus); masker condition (one masker deviant per stimulus); and combined condition (one target deviant and one masker deviant, at independent times, per stimulus). Each target deviant was the displacement of a target note (upward or downward) by two semitones from the target frequency. Each masker deviant was a single 500-ms time window in which all masker tones were elongated from 75 ms to 400 ms. The temporal location of the deviant (for both target and masker tasks) was randomly distributed along the 5.5-s trial duration, with timing as indicated by the behavioral buildup curve in Figure 4.

### Experimental Procedure

In the psychoacoustic experiment, participants were presented with 180 stimuli (three protection regions×four conditions×15 exemplars) per task. The progression from one trial to the next was initiated by the participant with a button-press.

In the MEG experiment, only the eight-semitone spectral protection region half-width was used, giving 60 stimuli (1 protection region × 4 conditions × 15 exemplars) per task. The interstimulus intervals (ISIs) were randomly chosen to be 1,800, 1,900, or 2,000 ms. For each task, the participants were presented with three blocks, repeating the ensemble of 60 stimuli three times (totaling 180 stimuli). Participants were allowed to rest after each block, but were required to stay still.

The identical stimulus ensemble (including identical ISIs in the MEG case) was presented for both target and masker tasks. Depending on the task being performed, participants were instructed to listen for the presence of a frequency deviant in the target rhythm (target task) or a duration deviant in the masker (masker task); each task deviant was present in exactly half the trials.

#### Psychoacoustical study

Participants were seated at a computer in a soundproof room. The signals were created offline and presented diotically through Sony MDR-V700 headphones. Participants controlled the computer using a Graphical User Interface (GUI) using the mouse. The task, as well as the basic use of the GUI, was described to participants. Participants were allowed to adjust the volume to a comfortable level before proceeding with the experiment.

A training block of 20 trials was presented before each task. In the target task training, the protection region half-width decreased from 12 semitones to four semitones in steps of four semitones. In the masker task training, it increased from four semitones to 12 semitones in steps of four semitones. Participants were permitted to listen to each sound as many times as desired; then participants were prompted to indicate whether a deviant was present. The correct answer was displayed afterwards. Participants pressed a button to initiate the presentation of the next stimulus.

Each participant performed both the masker task and the target task, with task order counterbalanced across participants. Each task required the participant to listen to the entire set of 180 stimuli described above. Each stimulus was presented only once, and no feedback was given after each trial. The entire session of both tasks lasted approximately 1.5 h.

#### MEG study

Participants were placed horizontally in a dimly lit magnetically shielded room (Yokogawa Electric Corporation). Stimuli were presented using Presentation software (Neurobehavioral Systems). The signals were delivered to the participants' ears with 50-Ω sound tubing (E-A-RTONE 3A; Etymotic Research), attached to E-A-RLINK foam plugs inserted into the ear canal, and presented at a comfortable loudness of approximately 70 dB SPL. The entire acoustic delivery system is equalized to give an approximately flat transfer function from 40–3,000 Hz.

Before the main experiment, a pre-experiment was run in which a 1-kHz, 50-ms tone pip was presented about 200 times. The ISI was randomized between 750 ms and 1,550 ms, and participants were instructed to count the tone pips. The aim of this task was to record the M100 response (a prominent peak approximately 100 ms after pip onset, also called N1m) used for differential source localization. The responses were checked to verify that the location and strength of neural signals fell within a normal range.

In the main experiment, participants were presented with three blocks of the 60 stimuli described above. Each participant performed both the masker task and the target task, with task order counterbalanced across participants. Participants were instructed to press a button held in the right hand as soon as they heard the appropriate deviant.

A training block with 12 sounds was presented before each task. Each training sound was presented twice. Participants verbally indicated the existence of the deviants and the feedback was given by the investigator. The entire session of both tasks lasted approximately 1 h.

MEG recordings were conducted using a 160-channel whole-head system (Kanazawa Institute of Technology). Its detection coils are arranged in a uniform array on a helmet-shaped surface on the bottom of the dewar, with about 25 mm between the centers of two adjacent 15.5-mm-diameter coils. Sensors are configured as first-order axial gradiometers with a baseline of 50 mm; their field sensitivities are 5 fT/√Hz or better in the white noise region. Three of the 160 channels are magnetometers separated from the others and used as reference channels in noise-filtering methods. The magnetic signals were bandpassed between 1 Hz and 200 Hz, notch filtered at 60 Hz, and sampled at the rate of *f_s_* = 1,000 Hz. All neural channels were denoised twice with a block least mean square (LMS) adaptive filter, first, using the three external reference channels [41], and second, using the two channels with the strongest cardiac artifacts [82].

### Data Analysis

#### Behavioral performance analysis

The ability of participants to perform the requested task was assessed by calculating a d-prime measure of performance [83]. For each condition (i.e., each task and protection region), we estimated the correct detection and false alarm probabilities for detecting the target or masker deviants; converted them to normal deviates (*z*-scores), and computed the d-prime value. The performance shown in Figure 1 depicts the mean d-prime values across participants. The error bars represent the standard error of mean.

To determine the effect of the target's tonal frequency on the neural responses, the stimuli were divided spectrally: each sound was characterized as a low- or high-frequency target tone sequence depending on the target tone's relation to the middle frequency 353 Hz (those with target tone frequency of 353 Hz were randomly assigned as low or high in such a way as to equipartition the high and low categories). A d-prime measure was then derived for each of the low or high target trials from both target and masker tasks.

To investigate the buildup of the target object during the target task, we divided the deviant trials according to the nine possible temporal locations of the deviant throughout the stimulus sequence. A probability of hit was then measured for each trial. Because of the temporal uncertainty in the false alarm trials, we calculated an average false alarm rate (irrespective of when the false response was issued), and combined it with the time-specific hit rate to derive a d-prime measure for each time segment. Using this behavioral assessment measure, five participants yielded nonpositive d-prime values due to their high false alarm rate and low hit rate, and were excluded from the analysis of buildup.

#### Neural data analysis

After recordings were completed and noise reduction algorithms applied, the responses to each stimulus, from 1.25 s to 5.5 s poststimulus, were extracted and concatenated, forming a single extended response with duration *T* = 765 s (4.25 s×60 sounds×three blocks) for each channel. This was done separately for each task block. The discrete Fourier transform (DFT) was applied on the single response, giving a single Fourier response from 0 to 500 Hz with frequency resolution of 1/765 Hz.

The evoked neural responses to the target sequences were characterized by the magnitude and phase of the frequency component at 4 Hz (the tone presentation rate) and were used for localization and for phasor maps. The complex magnetic field strength is given by the product of the value of DFT times the sampling interval (1/*f_s_*), and has units of fT/Hz. Power spectral density is calculated as the product of the inverse duration (1/*T*) times the modulus squared of the complex magnetic field strength, and has units of fT^2^/Hz. The remainder of the analysis was based on the normalized neural responses, defined to be the squared magnitude of the frequency component at 4 Hz divided by the average squared magnitude of the frequency components between 3 Hz and 5 Hz (excluding the component at 4 Hz), averaged over the 20 channels with the strongest normalized neural responses for each participant. The channels were allowed to vary from participant to participant to allow for inter-participant configuration variability. Using 10, 20, or 50 channels yielded similar findings; however, only the 20 channel analysis is reported here, indicating that this method is robust against the particular subset of channels used. This normalization is not biased by the task, since the average squared magnitude of the frequency components between 3 Hz and 5 Hz did not significantly differ between tasks.

The spatial pattern of the neural responses was represented by a phasor map, a graph of the complex (magnitude and phase) magnetic field on all channels. For each channel, the length of the vector arrow is proportional to the magnitude of the 4-Hz frequency component, and the direction of the arrow represents the phase according to standard polar coordinates. Red and green contours represent the magnetic field strength projected onto the line of constant phase that maximizes the projected field's variance [43]. The phasors are visually faded using the signal-to-noise ratio (SNR) of each channel as linear fading coefficients.

The normalized neural responses difference between target task and masker task was averaged across 14 participants to characterize attention gain effect. Furthermore, to evaluate the effect of attention at across frequencies, the same analysis is done at 4 Hz and the two adjacent frequency bins (4 Hz−Δ*f* and 4 Hz+Δ*f*), and also at 11 frequencies in the alpha, theta, and low gamma frequency bands, in approximately 5-Hz increments from approximately 5 Hz to approximately 55 Hz. For consistency with the frequencies examined in tests of phase coherence (next), we used Δ*f* = 1/(4.25) Hz, with analysis performed only at its integer multiples (e.g., 17Δ*f* = 4.0 Hz and 21Δ*f*≈4.94 Hz). The normalization is only weakly affected by the task, since the average squared magnitude of the frequency components did not vary strongly by task (no difference below 5 Hz, a relative enhancement in the target task of approximately 1 dB from 5 to 25 Hz, and a relative enhancement for the masker task of approximately 1 dB from 25 to 50 Hz).

To study attention modulation effects on the synchronization between two distinct neural populations, phase coherence between channels *m* and *n*, γ^2^ *_mn_*, is obtained from *Q = *180 trials [84],[85]:
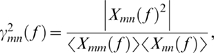
where *X_mn_*(*f*) is the average cross spectrum between channel *m* and channel *n*, *X_mm_*(*f*) is average power spectrum of the individual channel *m*:
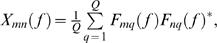
where 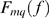 is the Fourier transform of the *q*th trial of channel *m* at frequency *f*. A coherence value of one indicates that the two channels maintain the same phase difference on every trial, whereas a coherence value near zero indicates a random phase difference across trials. The coherence difference between target task and masker task was computed for every channel pair. The standard error of the mean (SEM) ε*_mn_* was constructed to identify robust coherence change [84],[85]:
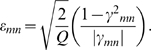



To emphasis phase modulation in auditory cortex, each participant's 20 channels with the strongest normalized neural response at target rate were included in further analysis. In addition, to exclude the artificial coherence that resulted from volume conduction effects on extracranial magnetic field and measure genuine phase correlation between distinct populations of neurons, only long-distance channel pairs (channel separation >100 mm) were included [85]. The difference between number of channel pairs with robust increased coherence and channel pairs with decreased coherence is normalized over the total number of long-range channel pairs for each participant. Furthermore, to evaluate the effect of attention at across frequencies, the same analysis is done at 4 Hz and the two adjacent frequency bins (4 Hz−Δ*f* and 4 Hz+Δ*f*), and also at 11 frequencies in the alpha, theta, and low gamma frequency bands, in approximately 5 Hz increments from approximately 5 Hz to approximately 55 Hz. For phase coherence (measured across trials), Δ*f* = 1/(4.25) Hz, and phase coherence was analyzed only at its integer multiples (e.g., 17Δ*f* = 4.0 Hz and 21Δ*f*≈4.94 Hz).

To investigate the possibility of hemispheric bias, the 20 channels with the strongest normalized neural response at the target rate were chosen from left and right hemispheres, respectively, to represent the overall neural activity of each hemisphere. Neural responses averaged across the 20 channels were subtracted across hemispheres for each task and for all 14 participants. Using 10, 20, or 60 channels yielded similar findings; however, only the 20-channel analysis is reported here.

To determine the effect of the tonal frequency on the neural responses, the stimuli were divided spectrally as described above. The neural responses at the target rate (both normalized response and phase coherence), from low- and high-frequency target tone stimuli, were obtained for each participant in the same way as described above, but with only appropriate epochs concatenated or phase-averaged.

To investigate the buildup of the target object in target task, the responses were divided temporally: the analysis epochs were divided into five temporal segments of 750-ms duration each, e.g., from 1.25 s to 2 s poststimulus (or 2 s to 2.75 s, etc.), were extracted and concatenated, forming a single extended response with duration *T* = 135 s (0.75 s × 60 sounds × 3 blocks) for each channel. The discrete Fourier transform (DFT) was applied on the single response, giving a single Fourier response of from 0 to 500 Hz with frequency resolution 1/135 Hz. The first segment began at 1,250-ms poststimulus since earlier time intervals showed substantial power at the frequency corresponding to the segment duration, an artifact indicating that the measured spectral power was extrinsic to the analysis window, not intrinsic to the neural signal. The segment duration of 750 ms was used since shorter durations did not show the buildup effect, an effect that is elaborated upon in the discussion of the quantitative model of neural buildup. An analogous analysis of phase coherence buildup over time was performed but did not yield significant results.

#### Behavioral versus neural correlation and bootstrap analysis

We correlated the effect of high versus low target frequencies in the behavioral and neural responses by contrasting the per-participant psychometric and neurometric measures. First, we scaled the neural data (i.e., the normalized responses to target) by a factor of three in order to match the absolute ranges of both neural and behavioral values. We then derived the angle (i.e., inverse tangent) of the slope relating the high versus low frequencies of the behavioral and neural data points for each participant and each task. The across-participant slopes were then combined using circular statistics to yield an angular mean for each task [86].

We performed a bootstrap procedure in order to confirm the positive (respectively, negative) correlation between the neurometric and psychometric functions in the target (respectively, masker) task. We followed a balanced bootstrap sampling procedure [87] by randomly selecting 14 participants with replacement and computing their angular sample mean and repeating this process 1,000 times. The procedure was controlled to ensure that all participants appeared the same number of times over all 1,000 bootstrap samplings. Confidence measures were then derived from the bootstrap statistics.

A similar statistical analysis was performed to correlate the psychometric and neurometric curves for the target detection buildup. To match the range of values from the neural and behavioral data, we scaled the neural responses by a factor of two (note that the different scaling is due to the reduced values of the normalized neural response due to the smaller window for the buildup analysis). The behavioral curves for each participant were then interpolated to match the sampling rate of the neural data. Subsequently, these two curves were then fitted by a first-order polynomial to derive the slope relating the two functions. The slope value was transformed into an angle and then combined across participants, following the same procedure described above. Note that the five participants with negative d-prime values were excluded from this correlation analysis, because of their questionable behavioral performance.

#### Neural source localization

Source localization for the M100 response was obtained by calculating the current-equivalent dipole best fitting the magnetic field configuration at the M100 peak, in each hemisphere. Source localization for the neural response to the target was obtained by calculating the complex current-equivalent dipole best fitting the complex magnetic field configuration at 4-Hz peak, in each hemisphere [43]. Only channels with SNR >4 were used in the fitting. Goodness of fit, as a function of the complex current-equivalent dipole, is given by one minus the residual variance ratio. As discussed in [43], the goodness of fit for complex magnetic field distributions and complex dipoles gives typical values that are much lower than for comparable real distributions, due to the doubling of number of degrees of freedom absorbing noise power. Significance of the relative displacement between the M100 and 4-Hz dipole sources were determined by a two-tailed paired *t*-test in each of three dimensions: lateral/medial, anterior/posterior, and superior/inferior.

### Quantitative Model of Neural Buildup

A model simulation to illustrate a mechanism of neural buildup was implemented in MATLAB (MathWorks). The model simulates MEG responses by generating 4-Hz signals whose phase is a random variable with constant mean, additionally corrupted by additive Gaussian white noise. This noise represents neural variability inherent in the neural processing mechanisms underlying the MEG signal and is critical to the model (external magnetic field noise had already been removed from the data by active filtering [41] and is not modeled). The normalized response power was calculated by the same method as in the experiment: concatenating 50 signals and normalizing the power at 4 Hz by the average power in the 3–5 Hz band, averaging the 20 best channels (out of 100 simulated auditory channels), and then averaging that over simulation runs. This was done for five different distributions of the phase random variable with standard deviations ranging from 1/60 to 1/12 of a cycle.

The model's level of Gaussian white noise was obtained by the biological requirement that the model's normalized response for the three-cycle window, with highest jitter, match the data's; the buildup rate as a function of decreased variability, as well as the average level of the normalized response for the one-cycle window, follow automatically. The simulated response results depend weakly on the number of channels simulated. The experimental MEG system records from 157 channels, but not all are strongly auditory. The results shown here use 100 simulated auditory channels.

## Abbreviations

MEG: magnetoencephalography
